# Advances in the Understanding of the Genetic Determinants of Congenital Heart Disease and Their Impact on Clinical Outcomes

**DOI:** 10.1161/JAHA.117.006906

**Published:** 2018-03-09

**Authors:** Mark W. Russell, Wendy K. Chung, Jonathan R. Kaltman, Thomas A. Miller

**Affiliations:** ^1^ Division of Pediatric Cardiology University of Michigan Ann Arbor MI; ^2^ Departments of Pediatrics and Medicine Columbia University New York NY; ^3^ Division of Cardiovascular Sciences National Heart, Lung, and Blood Institute Bethesda MD; ^4^ Department of Pediatrics University of Utah Salt Lake City UT

**Keywords:** clinical outcomes, congenital heart defects, genetics, Congenital Heart Disease, Genetics, Quality and Outcomes

## Introduction

Congenital heart defects (CHDs) are the most common type of birth defect occurring in ≈1% of live births^1^ and, if minor cardiac abnormalities such as bicuspid aortic valve are included, then the prevalence may be as high as 2% to 3%.^2^ Advances in surgical and perioperative care and catheter‐based interventions have dramatically improved survival, yet there continues to be ≈20% early mortality for the most complex cardiac defects.^3^ Furthermore, many of the survivors need long‐term medical care and have functional deficits in cognition, behavior, attention, and exercise performance that limit educational and employment opportunities and reduce their quality of life.^4^ As survival for patients with CHD has improved, there has been an increased emphasis on understanding variation in outcome and in improving short‐ and long‐term outcomes, which include but are not limited to survival. While recent efforts to optimize and standardize clinical practice and perioperative care have resulted in small incremental improvements, they have not led to major advances in clinical outcomes. Increasingly, the focus of outcomes research is on understanding the differences between individual patients (including genetic factors and specific variations in clinical care or clinical course) that predict or determine clinical outcomes.

Recently, the effort to better understand and improve clinical outcomes has been aided by complementary initiatives to identify the causes of CHD. A fall in the costs of high‐throughput DNA sequencing, advances in bioinformatic analyses, and an investment in funding the collection and genetic characterization of large cohorts of patients with CHD has rapidly advanced our understanding of the genetic architecture of CHD. What is emerging is an improved understanding of how underlying genetic factors can influence specific measured clinical outcomes and the importance of considering these factors when assessing the effectiveness of interventions and new treatment approaches. In this review, we will examine clinical outcomes such as survival, cognition and behavior, growth, and cardiac function for patients with CHD in the context of specific genetic etiologies.

## Common Outcomes Measures in CHD Patients

### Survival/Transplant‐Free Survival/Event‐Free Survival

Even with the improvement in postoperative survival for most types of CHD, survival rates remain an important clinical outcome for complex CHD for which early mortality can be as high as 20% and late mortality is a relatively common occurrence.^3^ Further improvements in survival will require a better understanding of patient‐specific risk factors that confer a higher risk for an adverse clinical outcome during the longitudinal management of CHD. Individual risk factors will also need to be categorized with respect to the timing of their impact on survival. Different mechanisms likely drive early, sometimes referred to as surgical or procedural mortality as opposed to late events. As more individuals with CHD survive into adulthood, the importance of understanding determinants of longitudinal survival increases. Clearly, genetic factors are an important contributor to differences between patients and, not surprisingly, genetic syndromes and nonsyndromic genetic variation have been noted to have a significant effect on long‐term survival after repair or palliation of CHD. Since cardiac transplantation is often used to rescue a patient who has failed surgical and medical management of their cardiac defect, patients who have required cardiac transplantation are often grouped with nonsurvivors to denote treatment failures. Since death and heart transplant are relatively infrequent occurrences, these outcomes will occasionally be grouped with major adverse events such as cardiac arrest, need for extracorporeal support, renal failure requiring dialysis, and other life‐threatening complications to yield an “event‐free” or “complication‐free” survival.

### Growth

Growth failure in CHD is a major and potentially modifiable comorbidity.^5^ In single‐ventricle populations, poor somatic growth is associated with prolonged hospitalization, decreased transplant‐free survival, and increased neurodevelopmental disabilities.^5^, ^6^, ^7^, ^8^, ^9^, ^10^ Poor somatic growth for a child with CHD begins in utero. The cause of poor fetal growth is likely multifactorial, involving genetic and placental risk factors along with abnormal regional blood flow and oxygenation.^11^, ^12^, ^13^, ^14^, ^15^ With an increased focus on somatic growth, nutritional interventions have become emphasized across many centers, including being a major focus of the National Pediatric Cardiology Quality Improvement Collaborative.^16^ Catch‐up weight gain is more achievable than attainment of normal length (or height).^17^, ^18^, ^19^ Lack of improvement in linear growth as well as the association between linear growth and neurodevelopmental outcomes^9^, ^10^ raises suspicion that a large portion of the variance in linear growth outcomes is driven by genetic predisposition, a suspicion supported by the association of pathogenic copy number variants (CNVs), linear growth, and poor neurocognitive outcomes.^20^

### Neurodevelopmental Performance

**Figure 1:**
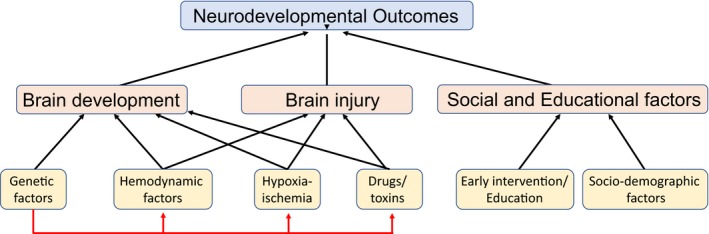
Factors affecting neurodevelopmental outcomes. Measured neurodevelopmental outcomes are directly influenced by how the brain has been formed and developed (brain development), whether or not it has been injured during development or perioperatively (brain injury), and how it has been affected by the patient's social and educational environment (social and educational factors). Genetic factors can have a primary effect on brain development. They can also have a secondary or modifying effect (red arrows) on other factors that affect brain structure and function, including hemodynamic factors, hypoxic/ischemic injury, and drug/toxin‐mediated effects.

As long‐term survival of CHD has dramatically improved, it is becoming increasingly evident that CHD survivors often have long‐term disabilities, including permanent neurodevelopmental (ND) deficits that can affect school performance, employability, and quality of life. The majority of patients with the most severe cardiac defects, such as complex single‐ventricle malformations, will have some degree of ND impairment and ≈15% to 30% will have severe cognitive and/or behavioral deficits. The causes of ND impairment in CHD patients are many and include developmental defects, abnormalities of the maternal‐fetal environment, and perioperative neurologic injury (Figure 1). Despite the identification of many covariates, combined, the known perioperative risk factors explain only ≈30% of the variance in ND outcomes, indicating that innate, patient‐specific genetic and physiologic factors may account for much of the variance.^21^ Genetic factors strongly influence brain development and contribute to the fetal response to the in utero environment and perioperative injury processes. What has made assessment of ND disabilities particularly challenging, in addition to the myriad of factors that can affect neurodevelopment, is the broad range of ND domains that can be affected and the fact that each of those domains and how they are best measured changes with age. One of the earliest measures that is commonly used is the Bayley Scales of Infant Development (BSID), which has been updated twice, most recently in 2006 (BSID‐III).^22^ The most recent version allows the assessment of ND performance in infancy across multiple domains including cognition, language, motor skills, social‐emotional function, and adaptive behavior. This proctored test can be supplemented with parent‐reported outcomes assessments such as the Ages and Stages Questionnaire (ASQ),^23^ which are well suited to ND follow‐up programs since they do not require an in‐person evaluation.

As patients age, ND assessments can be expanded to detect more subtle deficits in cognition and higher levels of reasoning and processing and to better characterize attention and behavior. Expanded ND assessments measure the following domains: academic performance, IQ testing, language skills, short‐term memory, attention and executive function, visual and spatial processing, fine motor skills, social skills, adaptive skills, and emotional/behavioral function. Previous studies have identified significant abnormalities in each of these domains in patients with CHD, although there is significant variability across patients and across CHD subtypes. Perhaps most prevalent have been attention deficit/hyperactivity disorders (ADHDs). A recent study evaluating 3552 CHD patients extracted from the National Health Insurance Research Database in Taiwan revealed an adjusted hazard ratio of 2.52 (95% confidence interval [CI], 1.96–3.2) for being diagnosed with ADHD and an adjusted hazard ratio of 1.97, (95% CI, 1.11–3.52) of being diagnosed with autism spectrum disorder compared to age/sex‐matched controls.^24^ The risks were even higher in subjects defined as having early developmental disorders. The risk may also vary by CHD subtype. A recent examination of 91 patients with tetralogy of Fallot demonstrated an ADHD prevalence of 39% and 19% in those with and without a genetic diagnosis, respectively, compared with 5% of controls.^25^ Of 111 patients with single‐ventricle CHD, 66% of patients received a psychiatric diagnosis, primarily anxiety disorder and ADHD, in long‐term follow‐up compared with 22% of controls.^26^ Although many studies looking at ND outcomes exclude individuals with extracardiac anomalies, when included, studies have consistently identified genetic factors as contributing to ND outcomes in patients with CHD. Of 321 survivors of single ventricle palliative repair who were evaluated at ≈14 months of age, genetic syndromes/anomalies were an independent risk factor for a lower‐than‐normative mental development score (MDI) on the BSID‐II assessment.^27^ In a study of 1770 subjects with a spectrum of CHD, the presence of genetic syndrome and/or extracardiac anomaly was similarly associated with an increased risk of a lower MDI and PDI (psychomotor developmental index) on the BSID‐II administered at 14 months of age.^28^ Taken together, these studies support the importance of assessing ND performance in CHD survivors and the significant impact that genetic factors have on ND measures.

### Ventricular Function

During operative repair or palliation of CHD, the heart is usually arrested and emptied to yield a bloodless operative field. The blood is circulated through a cardiopulmonary bypass machine, where it is filtered, oxygenated, and returned to the patient to perfuse all the organs and tissues including the heart. For some CHD surgeries, a period of complete circulatory arrest (no bypass flow) is required. Despite refinement of the technical approaches and the limitation of cardiopulmonary bypass and circulatory arrest times, injury to multiple organs and tissues including the heart occurs. This often results in a transient period of diminished ventricular function that, when pronounced, is referred to as low cardiac output syndrome.^29^ This diminished cardiac function can be associated with an increased complication rate and decreased event‐free postoperative survival.^30^ Sustained and progressive deficits in ventricular function can interfere with exercise performance, affect quality of life, and ultimately lead to heart failure, which may require heart transplantation. As with ND performance, cardiac function can be impaired in patients with CHD and can be caused by ischemia and ischemia‐reperfusion injury in the perioperative setting, mechanical injury during surgery (eg, attributable to ventriculotomy), or inherent genetically determined weaknesses and vulnerabilities. Systolic and diastolic ventricular function can be serially measured with echocardiographic or cardiac magnetic resonance imaging. Better delineation of genetic factors affecting ventricular function may aid the development of protective strategies and promote improved risk stratification.

## Genetic Architecture of CHD

Discussion of the impact of genetic factors on clinical outcomes begins with an understanding of the genetic architecture of CHD. Genetic contributors to CHD include disorders of chromosome copy number (eg, Down syndrome), subchromosomal deletions (eg, 22q11.2del) and duplications (chromosome 1p21dup), rare monogenic pathogenic variants, rare oligogenic deleterious variants, and common variants (reviewed by Zaidi and Brueckner^31^). Identification of the genetic causes of CHD has paralleled advances in genetic technologies. Aneuploidies, detected by karyotyping, were the first genetic variation associated with CHD. The trisomies (13, 18, and 21) and monosomies (Turner syndrome) along with large subchromosomal deletions (22q11.2), detected by fluorescent in situ hybridization and chromosomal microarray, make up the genetic etiology of 9% to 18% of CHD.^31^ Single gene etiologies, inherited in a Mendelian fashion, were initially detected by linkage analysis of large pedigrees. These genes were often transcription factors such as *TBX5, GATA4,* and *NKX2.5,* mutations of which likely explain a small percentage of CHD. Genome‐wide and high‐throughput sequencing technologies have enabled unbiased and thorough interrogation of the exome, the protein coding portion of the genome. Exome sequencing of probands and their unaffected parents have determined that ≈10% of CHD is caused by de novo (ie, not occurring in either parent) coding variants. If the CHD is accompanied by extracardiac anomalies and/or ND abnormalities, then de novo variants may explain ≈20% of disease.^32^ Pathogenic de novo variants typically occur in genes that are highly expressed in the developing heart and are enriched in certain biologic pathways such as chromatin remodeling, ciliary function, notch signaling, and sarcomere function. Single‐nucleotide polymorphism microarrays and novel analytic techniques of exome sequence data have detected rare, pathogenic CNVs) in ≈10% of patients with CHD.^33^, ^34^

**Figure 2:**
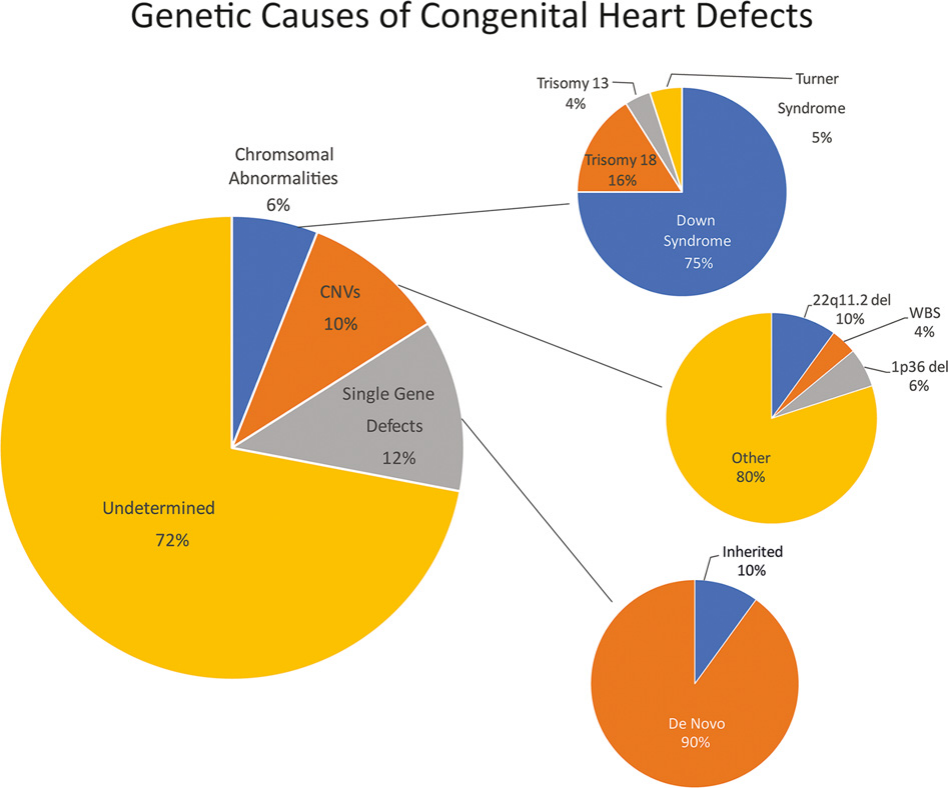
Genetic determinants of congenital heart defects. The majority of congenital heart defects do not have an identified genetic etiology. Unexplained CHD may be secondary to noncoding genetic, epigenetic, and environmental factors, among others. All estimates are approximate and are based on recent publications.^20^, ^31^, ^32^, ^33^, ^34^, ^35^, ^36^, ^37^, ^38^, ^39^, ^40^ CNVs indicates copy number variants.

A large percentage of CHD remains unsolved (Figure 2: pie chart of CHD causes).^20^, ^31^, ^32^, ^33^, ^34^, ^35^, ^36^, ^37^, ^38^, ^39^, ^40^ As larger numbers of exomes are sequenced, it is becoming apparent that rare, inherited variation plays a role, especially for isolated congenital heart disease.^35^ Other genetic mechanisms (including somatic mutation and multilocus variation) may have a role, as may epigenetic changes, noncoding variation, and environmental exposures.^31^

Each of these types of genetic variation can lead to abnormalities of cardiac development, resulting in CHD. In addition, concurrent developmental defects in other organs and tissues and associated deficits in resiliency or resistance to injury can lead to reduced survival and an increased rate of complications and comorbidities. The same genetic variation, therefore, can have pleiotropic effects and significantly impact clinical outcomes beyond the development of the structural heart disease. Progress in the understanding of the genetic determinants of CHD and their impact on clinical outcomes will be outlined in the subsequent sections.

### Chromosomal Abnormalities and CNVs

#### Abnormal chromosomal copy number

Abnormalities of chromosomal copy number, including the trisomies (13, 18, and 21) and monosomies (eg, Turner [45, X] syndrome), are commonly associated with CHD, with an incidence ranging from 80% to 90% for trisomy 13 and 18% to 50% for trisomy 21 (Down syndrome) and Turner syndrome.

##### Down syndrome (trisomy 21)

CHD is common in patients with Down syndrome, occurring in 40% to 50% of patients (see Table 1).^36^ Early surgical studies reported worse surgical outcomes in patients with Down syndrome undergoing repair for complete atrioventricular septal defect compared with patients without genetic syndromes.^41^, ^42^ More recently, several studies have demonstrated equal or decreased risk of in‐hospital mortality for patients with Down syndrome undergoing repair of CHD (including complete atrioventricular septal defect) compared with patients with normal karyotypes except among patients with single ventricle physiology.^43^, ^44^, ^45^, ^46^, ^47^, ^48^, ^49^ Several studies that included long‐term outcomes for complete atrioventricular septal defect repair have demonstrated a decreased rate of reoperation for left atrioventricular valve repair and for subaortic stenosis in patients with Down syndrome, which is likely related to valve and left ventricular outflow tract morphology differences.^46^, ^47^, ^49^

**Table 1:** Common Developmental Syndromes Associated With CHD

Condition/Diagnosis	Genetic Defect	Prevalence	Cardiac Defect	Associated Features
Down syndrome	Trisomy 21	1 in 1000 births	CAVSD, ASD, VSD, PDA, TOF	Cardiac defects (40–50%); short stature; cognitive deficits; atlantoaxial instability; immune system dysfunction; hypotonia; hypothyroidism
Turner syndrome	Monosomy X (may be mosaic; may involve all or part of X chromosome)	1 in 2000 to 5000 female births	CoA, BAV, Dilated Ao	Cardiac defects (≈30%); short stature (partially growth hormone responsive); cognitive deficits (usually mild) and ADHD; lymphedema
DiGeorge syndrome	22q11.2 del (most commonly)	1 in 4000 births	IAA, CAT, TOF	Cardiac defects (≈60–75%); short stature; cognitive deficits; thymic hypoplasia (leading to immune defects); hypocalemia/hypoparathyroidism
Williams‐Beuren syndrome	7q11.23	1 in 7500 births	supraAS, supraPS	Cardiac defects (75%); short stature; cognitive deficits; hypercalcemia; social personality; type 2 diabetes mellitus

ADHD indicates attention deficit/hyperactivity disorder; ASD, atrial septal defect; BAV, bicuspid aortic valve; CAT, truncus arteriosus; CAVSD, complete atrioventricular septal defect; CoA, coarctation of the aorta; dilated Ao, dilated ascending aorta; IAA, interrupted aortic arch; PDA, patent ductus arteriosus; supraAS and ‐PS, supravalvar aortic and pulmonary stenosis; TOF, tetralogy of Fallot; and VSD, ventricular septal defect.

One group of patients with Down syndrome that does have higher surgical risk is single‐ventricle palliation. Subgroup analysis demonstrated that among patients undergoing staged single‐ventricle palliation, patients with Down syndrome had higher in‐hospital mortality rates.^44^ A study from the Pediatric Cardiac Critical Care Consortium registry looking at all patients undergoing Fontan palliation confirmed a significantly increased mortality in patients with Down syndrome compared with those without, with most of these deaths occurring in the early postoperative period.^50^ This is thought to be attributable to the increased risk of pulmonary hypertension in these patients, which is not well tolerated in a single‐ventricle physiology.^51^

Although Down syndrome does not seem to confer an increased risk of mortality for most CHD repair, there have been studies showing that there is increased morbidity including significantly longer postoperative length of stay, increased risk of respiratory^52^, ^53^ and infectious complications,^46^, ^54^ pulmonary hypertension,^44^ higher rates of chylothorax,^44^ and increased risk of postoperative complete heart block.^44^, ^46^

##### Turner syndrome (45,X)

Turner syndrome is a common chromosomal condition caused by loss of part or all of the X chromosome in females. Short stature is common, as are ND deficits (see Table 1).^55^ Neurocognitive profiles in Turner syndrome can include a decrement in IQ of ≈10 to 15 points, learning disabilities, and challenges with executive function and ADHD. Because many individuals with Turner syndrome are mosaic, there is a wide range in severity of the associated clinical features. As with Down syndrome, patients with Turner syndrome have higher morbidity and mortality after surgical palliation of single‐ventricle heart disease compared with patients without chromosomal abnormalities.^56^, ^57^

#### Copy number variants

Copy number variants are large deletions or duplications of DNA that usually involve at least 100 000 base pairs of DNA but not the full chromosome. They can occur anywhere in the genome but often occur at sites bounded by regions of repeat or low‐complexity sequence that allow mismatches during DNA replication, resulting in duplication or loss of the intervening DNA sequence. CNVs can either be inherited or de novo. CNVs that are de novo, novel, oruncommon and are large are more likely to be disease causing or pathogenic. CNVs can involve one or more genes, and the resulting effects on clinical phenotype and clinical outcomes can depend on the number of genes involved and the roles of those genes in development of the heart and other organs and tissues.

##### 22q11.2 deletion syndrome

Recent population studies indicate that the 22q11.2 deletion is the most common microdeletion syndrome, occurring in 1 per 5950 live births^37^ and accounting for nearly 0.5% to 1.9% of all CHDs. Cardiac defects occur in 60% to 75% of cases with 22q11.2 microdeletion,^38^, ^39^ and there is an enhanced risk of CHD if there is a concurrent partial microduplication of the histone acetyltransferase complex member *KANSL1* on chromosome 17q21.31,^58^ highlighting the effect of genetic modifiers on clinical phenotype. The 22q11.2 deletion syndrome is commonly referred to as DiGeorge syndrome (DGS), although not all patients with DGS have the 22q11.2 deletion and not all individuals with the 22q11.2 deletion will display all the features of DGS (summarized in Table 1). As with CHD patients with larger chromosomal defects, growth, cognition, and behavior are all significantly impacted by the underlying genetic defect in patients with 22q11.2 deletions.

The presence of the 22q11.2 deletion also affects the survival and complication rate of CHD repair. Patients with the 22q11.2 deletion and/or a diagnosis of DGS have worse surgical outcomes, at least for certain types of CHD, including pulmonary atresia with ventricular septal defect and interrupted aortic arch.^59^, ^60^ The worse surgical outcomes appear to be in part due to more severe abnormalities of the pulmonary vasculature, with an increased incidence of multiple aortopulmonary collateral arteries and decreased arborization of the true pulmonary arteries.^61^ For patients with tetralogy of Fallot, those with 22q11.2 deletion required longer cardiopulmonary bypass times and a longer postoperative intensive care unit stay^62^ and had a worse quality of life on long‐term follow‐up.^63^ Associated immune defects require special handling of the blood products that are often required during the operation and in the perioperative setting, but severe complications such as graft‐versus‐host disease and overwhelming cytomegalovirus infection can be avoided by administering only CMV‐seronegative/irradiated blood products to patients with 22q11.2 deletion or DGS.^64^

##### Other major deletion/duplication syndromes

For most genes and CNVs, deletions are more clinically impactful than the corresponding duplication. In addition to the 22q11.2 microdeletion syndrome, other CNVs commonly associated with cardiac defects include microdeletion syndromes involving 7q11.23 (Williams‐Beuren syndrome), 1p36, and 8p23.

Williams‐Beuren syndrome (WBS) is a microdeletion syndrome affecting multiple genes on chromosome 7q11.23. It occurs in 1 in 7500 to 1 in 10 000 births and accounts for ≈0.25% of CHD, most commonly supravalvar aortic or pulmonary stenosis.^40^ WBS patients have growth deficiency that begins in utero and persists through childhood.^65^ Cognitive and behavioral deficits are common,^66^ and multiple organs and tissues can be affected (see Table 1).^67^ In addition, patients with WBS, in particular those with biventricular outflow tract obstruction and/or coronary ostial stenosis, are at risk for sudden death, especially when undergoing perioperative or periprocedural sedation, requiring careful anesthetic management and monitoring.^68^, ^69^ The risk of death is also present in patients with elastin arteriopathy (due to mutation or deletion of the elastin gene) in the absence of other features of WBS.

Other microdeletions and microduplications are also associated with CHD, and 2 additional CNVs occur often enough to be addressed specifically. Microdeletions of 1p36 occurs in 1 in 5000 births and are associated with abnormalities of cardiac structure (including patent ductus arteriosus, and atrial and ventricular septal defects) and/or function (specifically left ventricular noncompaction cardiomyopathy) in ≈70% of cases.^70^ Nearly all of those affected will have short stature and significant ND delay. Microdeletions of chromosome 8p23.1 are uncommon in the general population but can be found in a significant number of patients with CHD due to the loss of the *GATA4* gene, a transcription factor critical to heart development.^71^ In addition to cardiac defects, dysmorphic facies, short stature, and developmental delay are common features of 8p23.1 deletion syndrome.^72^

##### Rare and de novo CNVs

Pathogenic or potentially pathogenic CNVs have been determined to occur in ≈10% to 20% of patients with CHD.^20^, ^34^ While these commonly occur in patients with recognizable syndromes (such as DGS or WBS) and patients with dysmorphic features and/or multiple congenital anomalies, even nonsyndromic, nondysmorphic CHD patients are significantly more likely to harbor a potentially pathogenic CNV than individuals in the general population without CHD. In a series of 422 patients with nonsyndromic, isolated CHD (ie, no other anomalies), potentially pathogenic CNVs occurred in 12.1% of cases compared with 5% of healthy controls.^34^ Similarly, in a series of 223 patients with single‐ventricle cardiac defects, potentially pathogenic CNVs occurred in 13.9% compared with 4.4% of healthy controls.^20^ In a study of 2256 individual subjects with CHD, 283 parent‐child trios with CHD (tetralogy of Fallot) in the child, and 1538 controls, rare deletion CNVs (those occurring in <1% of the population at large) affected more genes and genes with higher haploinsufficiency scores (a measure of a gene's developmental intolerance of gene deletions) in CHD patients than in controls.^73^ Rare de novo CNVs occurred in 5% of the CHD trios, and several overlapping CNVs involved genes known to be involved in heart development including *HAND2* and *GJA5*, which encode for a cardiac transcription factor and gap junction protein, respectively.^73^ In that study, they were unable to detect a significant association of rare duplications with CHD, supporting the assertion that, in general, deletions more commonly have an impact on cardiac development. Mapping of overlapping, rare CNVs across multiple studies and identifying common critical regions facilitates identification of novel genes and signaling pathways involved in CHD pathogenesis.^33^, ^73^, ^74^

Given that pathogenic and potentially pathogenic CNVs can involve multiple adjacent genes and include genes critical to disease processes, it is perhaps not surprising that CNVs have been associated with multiple adverse outcomes in patients with CHD. As demonstrated for the CNVs associated with syndromic CHD, single‐ventricle‐type CHD patients with pathogenic CNVs have worse linear growth and neurodevelopmental performance (as determined by a lower Psychomotor Development Index score on the BSID‐II) at 14 months of age than those without CNVs.^20^ In a cohort of nonsyndromic patients with a broad range of heart defects requiring surgery before 6 months of age, presence of a potentially pathologic CNV was associated with a 2.6‐fold increased risk of death or transplant by 36 months post‐surgery.^34^ It is important to note that this study excluded all subjects with other significant congenital anomalies, indicating that the effect on transplant‐free survival was independent of any other known developmental abnormalities. Since pathogenic CNVs associated with CHD are distributed throughout the genome and involve a diverse set of genes, it will be important to identify the specific genes and signaling pathways associated with differential outcomes to develop protective and therapeutic strategies and improve risk assessment.

#### Single gene syndromes

##### RASopathies

The RASopathies are a group of autosomal‐dominant disorders with overlapping cardiac, growth, facial, and ND features caused by genes involved in the RAS mitogen‐activated protein kinase pathway. The spectrum of RASopathies includes Noonan syndrome (NS), cardiofaciocutaneous syndrome, Costello syndrome, and NS with multiple lentigines. Fifty percent of NS cases are explained by heterozygous *PTPN11* missense pathologic variants.^75^ An additional 30% can be explained by mutations in one of the RAS MAP kinase pathway genes including *SOS1, RAF1, RIT1, KRAS, SHOC2, NRAS*, SOS2, *BRAF, A2ML1, LZTR1, MYST4, RASA2, RRAS*,* SPRY1,* and *SYNGAP1*.^76^ NS and the other RASopathies share common features, including developmental delays, short stature, ptosis, hypertelorism, macrocephaly, and cardiac involvement (see Table 1).^77^, ^78^, ^79^, ^80^ Valvar pulmonary stenosis is a common form of CHD noted in patients with NS; however, NS patients with pulmonary stenosis are often not considered to be good candidates for balloon valvuloplasty due to the high rates of required reintervention (65%) after this procedure in the NS population.^81^

Coagulation factor deficiencies, thrombocytopenia, and platelet aggregation abnormalities have been reported,^82^ but are infrequently associated with postoperative bleeding complications (<2% of individuals).^83^ Lymphatic abnormalities are common, and chylous effusion is a regularly reported complication of cardiac surgery. Renal anomalies including vesicoureteral reflux, hydronephrosis, and dysplastic kidney are seen in 10% to 20% of individuals.^84^

##### Ciliopathies

Ciliopathies are due to abnormal cilia structure and function and are associated with heterotaxy and a range of genetic syndromes including Bardet‐Biedl syndrome, Alstrom syndrome, McKusick‐Kaufman syndrome, and Ellis van Creveld syndrome. The associated clinical features vary by condition.

Heterotaxy is associated with CHD in 50% to 95% of cases and can be associated with almost any type of CHD, but the most common defect is an atrioventricular canal defect that is frequently unbalanced.^85^ Heterotaxy can be associated with complete situs inversus, left atrial isomerism (polysplenia), and right atrial isomerism (asplenia). Abnormalities of spleen number (asplenia or polysplenia) may result in functional asplenia with increased susceptibility to infection. Gut malrotation poses a risk for volvulus. Extrahepatic biliary atresia is a significant extracardiac complication that increases mortality. As many as 37% of heterotaxy patients may have primary ciliary dyskinesia, which is associated with chronic productive cough, rhinitis, sinusitis, otitis media, bronchitis, and bronchiectasis.^86^ Poor mucociliary clearance leads to infection and inflammation of the airway and may contribute to the higher frequency of respiratory complications in patients with ciliary dysfunction.^87^ Cognition and intellectual function are usually normal.

Syndromic sensory ciliopathies are caused by abnormalities in the sensory or signaling functions of cilia and are commonly associated with defects in the eyes, ears, skeleton, brain, kidney, and liver in addition to CHD that includes situs abnormalities, atrioventricular canal defects, septal defects, and valve defects.^88^, ^89^, ^90^, ^91^, ^92^, ^93^ Common features include retinitis pigmentosa, cone‐rod dystrophy, sensorineural hearing loss, and brain malformations including brain stem malformations (molar tooth sign), Dandy‐Walker malformation, neural tube defects including encephalocele, holoprosencephaly, and agenesis of the corpus callosum. Many individuals with syndromic sensory ciliopathies are developmentally delayed or intellectually disabled. Obesity and diabetes mellitus are common. Skeletal anomalies can be associated with short stature, thoracic dysplasia, short limbs, and polydactyly. Hepatic fibrosis, hepatic cysts, polycystic kidneys, and nephronophthisis are observed with many of the conditions.

##### Chromatin modifiers

Initial studies in families affected by heritable congenital cardiac defects identified mutations in cardiac transcription factors such as *NKX2‐5, GATA4, TBX5, TBX1,* and *TBX20* as important causes of CHD. For some of these transcription factors, the effects were limited to the heart, which is where they are primarily expressed. Other cardiac transcription factor mutations, such as those involving *TBX5* (associated with Holt‐Oram syndrome) and *TBX1* (associated with some features of DGS), have major extracardiac manifestations but are not associated with known differences in clinical outcomes. Perhaps the most important cardiac complication of transcription factor mutations is disruption of the cardiac conduction system, which can lead to complete heart block in some individuals with *NKX2‐5* and *TBX5* mutations.^94^, ^95^

However, regulators of the transcriptional machinery, such as those that modify chromatin architecture by altering histone structure and function through acetylation, methylation, phosphorylation, and ubiquitination, are often more broadly expressed and, when mutated, can affect the development of multiple organs and tissues in a manner that directly impacts clinical outcomes. Mutations of the chromatin modifiers, *KMT2D* and *KDM6A,* which encode for lysine (K)‐specific methyltransferase 2D and lysine‐specific demethylase 6A, cause Kabuki syndrome, a developmental disorder affecting the heart, brain, urogenital system, craniofacial structures, and linear growth (height). Heart defects, which can range from mild (atrial septal defect, ventricular septal defect, patent ductus arteriosus, coarctation of the aorta) to more severe (tetralogy of Fallot, single‐ventricle CHD), occur in 31% to 58% of Kabuki syndrome patients.^96^, ^97^ Observed cardiac defects often involve left ventricular outflow tract obstruction, including Shone complex and hypoplastic left heart syndrome (HLHS). In a recent study performed by the Pediatric Cardiovascular Genomics Consortium (PCGC) of 362 cases of critical congenital cardiac defects including 60 patients with HLHS, de novo mutations were noted in 8 genes involved in the regulation of methylation of histone H3, lysine 4 (H3K4),^98^ including *KMT2D* (associated with Kabuki syndrome); *CHD7* (associated with CHARGE syndrome); *KDM5A* and *KDM5B* (H3K4 demethylases); *WDR5*, and *RNF20*,* UBE2B,* and *USP44,* which are involved in histone ubiquitination. Mutations were also noted in *SMAD2,* which is involved in signaling in the embryonic left‐right organizer through demethylation of H3K27. In this study, the patients with mutations involving histone‐modifying genes had a higher incidence of extracardiac manifestations including developmental delay and short stature.

#### Single gene (nonsyndrome)

##### De novo variants

Exome sequencing analysis of the PCGC cohort has demonstrated that ≈10% of CHD can be explained by de novo single‐nucleotide variants. When the cohort is parsed by associated abnormalities, de novo variants in genes highly expressed in the heart contribute to 10% of CHD associated with extracardiac anomalies, 6% of CHD with ND abnormalities, and 20% of CHD associated with both extracardiac and ND abnormalities.^32^ These findings suggest a pleiotropic effect of many of these de novo mutations.

The extracardiac abnormalities found in the PCGC cohort are wide ranging and affect many different organ systems, including craniofacial, pulmonary, gastrointestinal, orthopedic, and genitourinary, among others. Patients with CHD and extracardiac abnormalities are at increased risk of mortality due to increased complexity of care, increased risk of cardiac surgery, and additional sources of potential morbidity and mortality.^99^, ^100^

There was significant overlap between the genes with de novo mutations found in the PCGC cohort and genes with de novo mutations found in cohorts of patients ascertained for neurodevelopmental phenotypes. These overlapping genes are typically expressed in both the developing heart and brain. CHD patients with damaging de novo mutations found in these overlapping genes have an absolute risk of >70% of having ND abnormalities.^32^ Of the groups of genes identified, damaging mutations in the chromatin modifier genes had the highest risk of conferring a ND abnormality phenotype. These findings are significant because they provide a causal genetic link between CHD and ND abnormalities and imply that specific genotypes may strongly predict future ND outcome. They also have potential clinical implications. It is possible to imagine a clinical genetic test that can identify patients at particularly high risk of poor ND outcomes to target for neuroprotective measures and early childhood surveillance and intervention.

##### Structural proteins

While more commonly associated with cardiomyopathy (dilated, hypertrophic, or restrictive), mutations in genes encoding for components of the cardiac sarcomere, the basic contractile unit of striated muscle, have been determined to be responsible for familial and sporadic CHD. Examples include mutations in *MYH7* (β myosin heavy chain) in individuals with Ebstein anomaly of the tricuspid valve, in *ACTC1* (cardiac α actin) in familial ASD, and in *MYH6* (α myosin heavy chain 6) in autosomal dominant familial ASD and sporadic cases of more complex CHD, including Shone complex and HLHS.^35^ There is mounting evidence that genetic variation in sarcomeric genes can concurrently cause CHD and affect ventricular function. Mutations in *MYH7* that cause Ebstein anomaly also lead to ventricular noncompaction and reduced ventricular function.^101^ Similarly, multiple studies have shown that CHD patients with sarcomeric mutations have differential clinical outcomes, including reduced ventricular performance and transplant‐free survival. In a recent study of 2645 parent‐offspring trios and 226 singletons who underwent exome sequencing by the PCGC, 7 had recessive genotypes involving *MYH6*.^35^ Five of the 7 had left ventricular outflow tract obstructive lesions, including 4 with Shone complex (which is characterized by mitral and aortic valve abnormalities). Abnormal ventricular function was noted in 4 of the 7 subjects with *MYH6* mutations. Reduced ejection fraction, a measure of systolic ventricular function, was also noted in 2 subjects with HLHS who had recessive *MYH6* mutations.^102^ A case‐control study of 190 patients with HLHS noted an increased burden of damaging *MYH6* variants in HLHS cases versus 1000 Genomes Project controls and reduced transplant‐free survival in HLHS patients with *MYH6* mutations compared with other HLHS patients.^103^ The differential survival was potentially due to impaired cardiomyogenesis and to dysregulation of genes related to myocardial structure and function. Collectively, these studies demonstrate the increasingly recognized role of sarcomeric genes in the pathogenesis of CHD and the important effect that sarcomere gene mutations have on ventricular function and long‐term survival.

## Genetic Modifiers of Clinical Outcomes

In addition to rare and de novo DNA sequence variants that can affect developmental pathways directing morphogenesis of the heart and other organs and tissues, more common genetic variants (which may not have any clinical effect under normal conditions) may lead to important differences in treatment responses and be important modifiers of clinical outcomes. Multiple clinical outcomes in patients with CHD, including survival, ND performance, and ventricular remodeling and function, have been demonstrated to be in part dependent on common genetic variants.

Perhaps the best described of these common genetic variants involves the ND effects of the different alleles of apolipoprotein E (ApoE) in patients with CHD. ApoE is a lipoprotein that is a primary cholesterol transporter in the central nervous system.^104^ It is produced by astrocytes and transports cholesterol to surrounding neurons. Its fundamental role in a wide range of neurologic conditions has been well described,^105^, ^106^, ^107^ and it appears to be an important regulator of neuronal homeostasis and resistance to injury. There are multiple isotypes of ApoE (ε2, ε3, ε4) with different functional properties. Individuals with at least 1 copy of the ε4 allele are at higher risk of Alzheimer disease^108^ and worse outcomes after traumatic brain injury.^109^ In patients with CHD, the ApoE ε2 allele is associated with worse early ND performance in patients with CHD,^110^ a deficit that persists as patients age^111^ and that has been replicated in a similar but distinct patient cohort.^112^ It has been proposed that ApoE allele status affects neuroresiliency and that the ApoE ε2 allele renders patients less resistant to neuroinjury that may occur in utero or perioperatively in patients with CHD.

Ventricular remodeling and function and postoperative survival in CHD has also been determined to be in part dependent on common genetic variants. Genetic variants associated with increased activation of the renin‐angiotensin‐aldosterone system were determined to be associated with multiple outcomes, including the reverse remodeling that occurs after the second‐stage palliative surgery for patients with single‐ventricle CHD^113^ and is associated with impaired diastolic function after the third stage of repair for single‐ventricle CHD, the Fontan operation.^114^ A vascular endothelial growth factor A allele linked to enhanced vascular endothelial growth factor A expression was associated with preserved ventricular function^115^ and postoperative survival^116^ in patients requiring CHD surgery in infancy. Lower event‐free survival has also been associated with adrenergic signaling pathway variants that increase catecholamine release or sensitivity in patients with single‐ventricle CHD.^117^

## Implications for Clinical Care/Outcomes Improvement/Future Research

### Perioperative Management

With an increased understanding of how genetic factors affect clinical outcomes (summarized in Table 2), there will be opportunities to target therapies to the specific needs of each individual patient. Currently, the most important role for preoperative genetic testing is in the CHD patient with possible 22q11.2 deletion syndrome. As noted above, patients with 22q11.2 deletion syndrome have thymic hypoplasia, which requires special handling of blood products before transfusion or exposure during cardiopulmonary bypass. Since clinical features of 22q11.2 deletion syndrome may not be apparent, especially in infants, testing for 22q11.2 deletion should be performed by fluorescent in situ hybridization, multiplex ligation‐dependent probe amplification assay, or quantitative polymerase chain reaction. Alternatively, chromosomal microarray testing can detect microdeletions and duplications anywhere throughout the genome. In addition to special handling of blood products, serum calcium levels need to be closely monitored and repleted as needed. The differential outcomes in subjects with 22q11.2 deletion and pulmonary atresia/ventricular septal defect may be primarily related to differences in vascular anatomy and may not require additional considerations for the genetic etiology beyond that required to address the more complex anatomy. Similarly, specific anatomic features such as coronary ostial abnormalities and biventricular outflow obstruction place patients with elastin arteriopathy (including those with WBS) at risk for sudden death, requiring cautious anesthetic management.

**Table 2:** Impact of Major Categories of Genetic Determinants of CHD and Their Effects on Selected Clinical Outcomes

Type of Genetic Variation	Outcome Domain				
	Survival	ND	Growth	V Function	Notes
Chromosomal abnormality					
Down syndrome	+/−a	++	+++	−	Higher mortality for single V heart defects; other defects unaffected^a^
Trisomy 18	++	++++	++++	−	
Trisomy 13	+++	++++	++++	−	
Turner syndrome	−	−	+++	−	
CNV					
22q11.2	+/−a	+	++	−	Higher mortality for pulmonary atresia with VSD; other defects unaffected^a^
Williams syndrome	+	+	++	−	
1p36 del	+	+	+	−	
Others	+	+	+	−	
Single gene disorders (rare variant)					
RASopathies	+/−a	− to ++	+	−	Higher mortality in cases with severe, early HCM^a^
Ciliary defects	−	−	−	−	Increased respiratory complications
Transcription factor	−	−	−	−	
Chromatin remodeling	−	+	+	−	
Sarcomeric	−	−	−	++	
Single gene disorders (common variant)					
ApoE (e2 allele)	−	+	−	−	
RAAS pathway	−	−	−	+^a^	Effect on ventricular remodeling in single V heart disease*
VEGFA variant	+	−	−	+	
Adrenergic signal	+	−	−	−	

Magnitude of effect represented by the number of +. No known effect represented by −. Outcomes include survival, neurodevelopment (ND), growth and ventricular (V) function. ApoE indicates apolipoprotein E; CNV, copy number variant; HCM, hypertrophic cardiomyopathy; RAAS, renin‐angiotensin‐aldosterone system; single V, single‐ventricle; VSD, ventricular septal defect; and VEGFA, vascular endothelial growth factor A.

a indicates that there is a explanation of the score in the notes for that outcome.

Another scenario in which differential clinical outcomes requires careful consideration of surgical approach and treatment plan involves the trisomy syndromes, including trisomy 13, 18, and 21. As noted above, low survival rates for patients with trisomy 21 and single‐ventricle cardiac defects (or trisomy 13 or 18 and any cardiac defect) has led many institutions to advise against palliative intervention in those cases.

Future improvements in perioperative and longitudinal care practices may rely in part on an improved understanding of individual factors, both genetic and nongenetic (ie, related to patient age, sex, medical history, and other health and treatment factors), that affect treatment response and clinical outcomes. Some of these will be related to pharmacogenomic factors, which affect a patient's biologic response to specific drugs. The studies examining the effects of renin‐angiotensin‐aldosterone system pathway genetic variation (and targeting of that pathway with angiotensin‐converting enzyme inhibition)^113^, ^114^ as well those studies assessing the effect of adrenergic pathway variation on clinical outcomes^117^ in patients with CHD indicate that therapeutic approaches tailored to specific genetic profiles may help improve outcomes. This type of precision medicine approach has been applied in other medical settings and is just beginning to be considered for the care of patients with CHD.

### Outcomes Assessment/Improvement

Perhaps the most immediate implication of the improved understanding of the impact of genetic factors on clinical outcome measures in patients with CHD is the need to account for those factors in outcomes research and analyses. As noted above, genetic factors can affect multiple outcomes measures (including neurodevelopment, growth, ventricular function, and survival), with effects that range from rare to common in prevalence and from mild to substantial in severity. While randomization may be able to distribute genetic factors between treatment groups in large trials, failure to account for important genetic determinants to specific outcomes measures may mask or dilute important treatment effects if the genetic effect is an unmeasured confounder of the treatment. As genetic determinants of CHD outcomes become better defined, it may be possible to stratify subjects by genetic risk for specific outcomes to identify different subpopulations responsive or resistant to the treatment or intervention.

#### ND performance

Cognition and higher‐level processing, motor function, and behavior and attention can all be significantly affected by genetic factors in patients with CHD. Therefore, studies assessing for the effectiveness of therapeutic interventions on ND outcomes in patients with CHD should ideally be structured to account for important genetic determinants in the analysis. It will be important to determine if specific types of genetic differences are equally distributed between the treatment groups and between treatment responders and nonresponders. It may be that the effectiveness of specific interventions designed to promote neurodevelopment may be less effective in those subjects with certain genetic features, and their inclusion in a batch analysis may obscure the effectiveness of the intervention in other patients.

Work to date suggests that just eliminating from the analysis those subjects with recognizable syndromes may not be sufficient to account for significant genetic effects on ND performance measures. Genomic characterization (chromosomal microarray analysis) and exome/genome sequencing of nonsyndromic CHD subjects has determined that pathogenic CNVs and mutations in genes responsible for both heart and brain development occur with sufficient frequency and have a significant enough impact to merit consideration when assessing ND performance in patients with CHD. Recent trials have sought to better understand ND deficits using anatomic and functional neuroimaging and to improve ND outcomes using early intervention strategies. Including in these studies patients who have undergone detailed genomic characterization will improve our understanding of how genetic factors influence brain structure and organization and affect ND performance and the response to intervention. We anticipate, based on the work to date, that genetically determined deficits will affect different ND domains and will be best accommodated by ND domain−specific and/or genetic mechanism‐specific interventions. Similarly, the effectiveness of any ND intervention will be best assessed with respect to any underlying genetic susceptibility.

#### Growth

As noted above, catch‐up weight gain is more achievable than maintenance of normal length.^17^, ^18^, ^19^ As a result, practices aimed at improving neonatal and infant growth may be responsible for the increased incidence of abnormal body mass index now reported in adolescents with CHD. While disease‐specific growth curves are available, and commonly used in clinical practice for some genetic syndromes (such as trisomy 21), the adjustment for growth potential based on less common genetic variations is not readily available. While many clinicians may base caloric strategies on proportional growth, better understanding of the genetic impact on growth potential will allow for a more personalized approach in many high‐risk infants whose caloric intake is not self‐regulated. Furthermore, similar to the need to control genetic risk in ND studies, research aimed at improving growth and minimizing associated comorbidities may currently be confounded by the inability to appropriately stratify treatment arms based on their true growth potential.

#### Ventricular function

The impact of genetic variation on ventricular function in patients with CHD is not yet well understood. Clearly, there are common genetic variants (eg, vascular endothelial growth factor A rs833069) that can have a modest impact on ventricular function^115^, ^117^ and rare genetic variants (eg, selected *MYH6* variants) that can have a more significant impact.^102^ There are potentially 2 important implications of the findings to date. First, it is important to note that there are an increasing number of examples in which patients with CHD have a mutation that affects a gene that can also cause ventricular dysfunction and dilated cardiomyopathy. While this may affect only a small percentage of patients with CHD, it may be important to consider genetic testing for concurrent dilated cardiomyopathy in a CHD patient with a decline in ventricular function that is out of proportion to the cardiac lesion or its treatment. Second, studies evaluating the impact of common genetic variation on ventricular structure and function^113^, ^114^, ^117^ suggest that variation in specific signaling pathways such as the renin‐angiotensin‐aldosterone system or adrenergic signaling may be suitable for pharmacologic targeting to help improve ventricular function, ventricular remodeling, and even survival in all CHD patients or in selected patients with genetic predisposition to over‐ or underactivation of those pathways. Ongoing studies examining ventricular function (in both a longitudinal and cross‐sectional manner) in CHD subjects who have had genomic characterization with exome or genome sequencing will likely identify novel mediators of ventricular function in CHD patients and help assess the relative impact of genetic variation on clinical outcomes related to ventricular performance.

#### Survival

Different mechanisms likely affect early peri‐operative survival compared with long‐term survival. To date, genetic determinants such as the presence of a pathogenic CNV or inherited variants in specific signaling pathways primarily affect mid‐ and long‐term survival after surgery for CHD in infancy. As these genetic determinants of long‐term outcomes become validated and better defined, it may be possible to adapt longitudinal follow‐up and institute compensatory pharmacotherapy to help modify and improve outcomes, especially in those at highest risk. Identification of the genetic determinants of early outcomes has been more challenging likely because of the large effects of technical surgical factors and patient‐specific complications. It is anticipated that early outcomes, like mid‐ and late outcomes, will be modified by specific genetic factors, the identification of which may depend on more precise determination of the vulnerable or fragile patient that requires escalation of care to prevent morbidities and mortality.

#### Future directions

Increasingly robust documentation and tracking of short‐ and long‐term outcomes combined with more widespread clinical and research‐based genetic characterization of CHD patients promises to lead to rapid advances in the application of precision medicine approaches to the care of patients with CHD. Linkage of information across different data sources, including genetic, surgical, and perioperative, and longitudinal follow‐up data sets, will help identify genetic patterns leading to adverse clinical outcomes and foster the development of individualized care and follow‐up programs tailored to the genetic strengths and vulnerabilities of each patient. Challenges will include (1) the storage, processing, and analysis of large amounts of data; (2) the adjudication of variants as pathogenic, likely pathogenic, or unknown significance (along with real‐time updating of status based on accumulating evidence); (3) the assignment of relative contributions of specific genetic factors to each outcome; and (4) the maintenance of privacy protections as information is shared across platforms and continually updated.

## Summary

Rapid advances in the identification of the genetic determinants of the causes of CHD coupled with the linkage of genetic testing and clinical outcomes data has allowed substantial improvement in our understanding of how genetic variation affects clinical outcomes in patients with CHD. What is emerging is that clinical outcomes in patients with CHD are dependent on a combination of disease‐specific, treatment‐related, and individual patient‐specific factors. Underlying genetic variation has an increasingly recognized important impact on outcome measures, including neurodevelopment, growth, ventricular function, and survival. Our ability to accurately assess outcomes in patients with CHD and to design and evaluate intervention strategies will depend on a continued increase in our understanding of the relative impact of each outcome determinant, including genetic determinants. In time, this will hopefully lead to a precision medicine type of approach in which best clinical practices are modified to optimally meet the needs of each individual patient, resulting in improved care and better clinical outcomes.

## Sources of Funding

This work was supported by the National Heart, Lung, and Blood Institute (U01) HL098163 (Dr Chung). The views expressed in this manuscript are those of the authors and not necessarily those of the National Heart, Lung, and Blood Institute or the National Institutes of Health

## Disclosures

None.
